# A simple modified technique for screw fixation of displaced intra-articular calcaneus fracture through a sinus tarsi approach: a comparison with plate fixation

**DOI:** 10.1186/s12891-024-07873-5

**Published:** 2024-09-18

**Authors:** Mohammad Reza Bahaeddini, Arian Rahimi Konjkav, Amir Aminian, Pouria Tabrizian, Sajad Noori Gravand, Shayan Amiri, Mohammad Sadegh Mirjalily, Hamed Tayyebi, Farid Najd Mazhar

**Affiliations:** 1 Department of Orthopedics, School of Medicine, Iran University of Medical Sciences, Tehran, Iran; 2 Shafa Yahyaeian Orthopedic Hospital, Baharestan Square, Tehran, 1157637131 Iran

**Keywords:** Displaced intra-articular calcaneus fracture, Sinus tarsi approach, Plate, Screw, Fixation

## Abstract

**Background:**

Plates and screws are frequently used for the fixation of displaced intra-articular calcaneus fracture (DIACF). In this study, we compared the outcomes of a modified screw fixation technique with plate fixation via a sinus tarsi approach (STA).

**Methods:**

A series of 187 DIACF patients who were treated via an STA using a plate fixation (*n* = 81) or a screw fixation (*n* = 106) were included. Screw fixation was done with two 2.7 mm screws and two 6.5 mm cannulated screws. Outcomes were evaluated radiographically and clinically. Clinical evaluations included pain assessment by Visual Analogue Scale (VAS) and functional assessment by the American Orthopaedic Foot and Ankle Society (AOFAS) questionnaire and Foot Function Index (FFI).

**Results:**

The mean final VAS was smaller in the screw group (*P* = 0.01). The mean AOFAS and FFI scores were not significantly different between the two groups (*P* = 0.17 and *P* = 0. 19, respectively). The mean improvement of Bohler’s angle, but not the Gissane’s angle, was significantly greater in the screw group (*P* = 0.014 and *P* = 0.09, respectively). The mean improvement of calcaneal length and height were not significantly different between the two groups (*P* = 0.78 and *P* = 0.22, respectively). The hardware removal rate was 14.8% in the plate group and 3.8% in the screw group (*P* = 0.007).

**Conclusion:**

The modified screw fixation method provides lower pain, better radiographic outcome, and lower rate of hardware removal compared to plate fixation in the treatment of DIACF.

## Introduction

Calcaneus fractures are infrequent injuries accounting for 1–2% of all human fractures [1, 2] and can be extra-articular or intra-articular [1]. Displaced intra-articular calcaneal fractures (DIACFs) comprise up to 75% of the calcaneal fractures and may cause hindfoot deformity, leading to long-term pain, stiffness, and disability [3, 4].

Treatment of DIACFs remains challenging [5]. Although various therapeutic options have been used in the past, the clinical outcomes of these procedures have been mostly unsatisfactory [5]. With an improved understanding of DIACFs, open reduction and internal fixation (ORIF) is now recommended as the standard treatment for these fractures, which can be done through various approaches [6]. Amongst, a sinus tarsi approach (STA) uses a small incision that allows the articular reduction with a limited soft tissue dissection, thereby causing lower complications [5, 7, 8].

Several devices are available for the fixation of calcaneal fracture following the reduction of DIACFs through an STA, including plate, Kirschner wires, and screws [9]. Whatever device is selected, painful hardware removal remains the most common STA complication in the long term [9, 10]. Hence, identifying a fixation device with lower rates of implant removal after reduction is of considerable importance.

Screws have recently attracted attention in the fixation of DIACF, and its outcomes alone or in comparison with plate have been reported in several studies. Theoretically, the incision made for screw fixation is smaller than the plate. In addition, screws are smaller than plates and, therefore, are associated with less damage to the nerves, vessels, and tendons and, therefore, less need for early removal. Nevertheless, screw fixation is not as strong as plating and could be associated with a higher rate of fracture displacement [11]. We aimed to augment the DIACF fixation by using a modified pattern for screw placement. At the same time, we reduced the number of screws, as the smaller number of screws provides less irritation and hence, less need for future removal. In this study, we aimed to compare the outcomes and complications of this modified screw fixation technique with plate fixation in DIACF patients who were treated by an STA.

## Patients & methods

This cohort study was approved by the review board of our institute under the code IR.IUMS.FMD.REC.1398.344. Medical profiles of patients with a calcaneal fracture who underwent surgical treatment in our tertiary orthopedic hospital between 2018 and 2022 were retrospectively reviewed. Inclusion criteria were unilateral close fracture, DIACF type II and III according to the Sanders classification [12], treatment through an STA, fixation of the fracture with a plate or screw, and a minimum follow-up of 12 months. Exclusion criteria were a prior history of fracture or surgery in the involved calcaneus and any deformity in the lower limb. Finally, 187 patients who met the study criteria were included in the analysis. The fracture fixation was made with a plate in 81 (43.3%) patients and with a screw in 106 (56.7%) patients.

### Surgical procedure

**Fig. 1:**
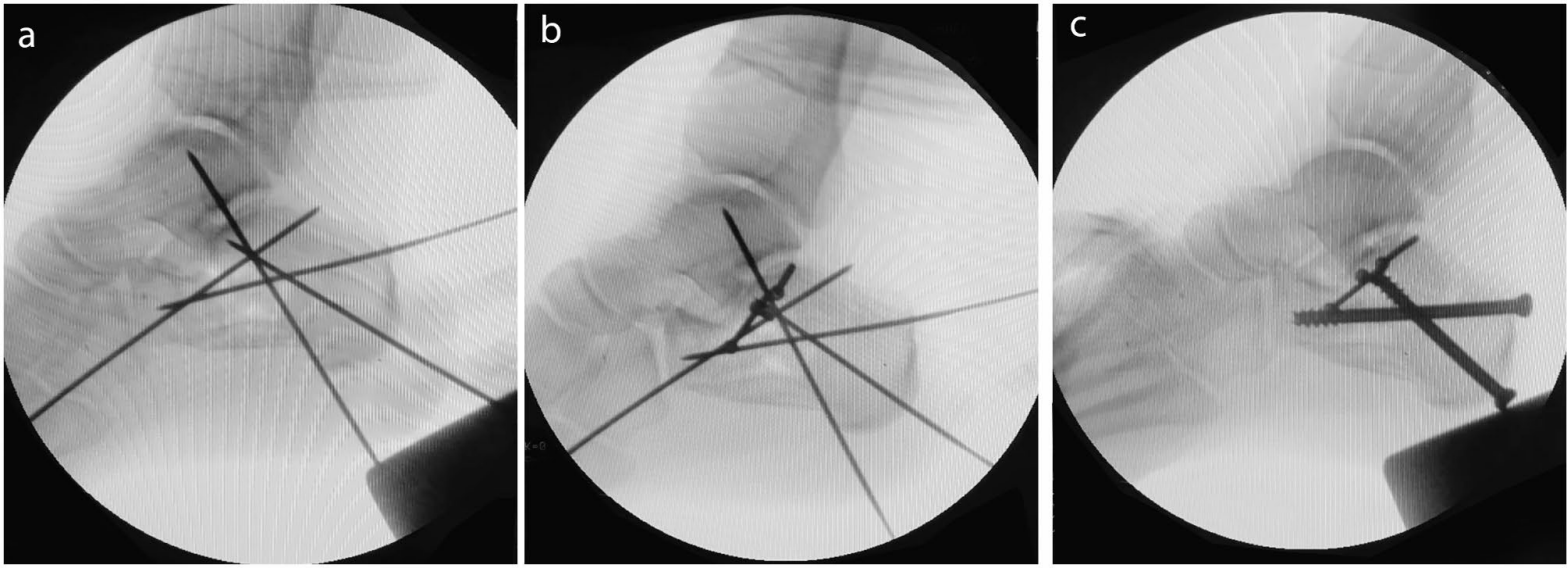
Intraoperative Lateral C-arm imaging showing (**a**) calcaneus fracture reduction and temporarily fixation with pin; (**b**) lag of posterior facet fragments with 2.7 mm screws and restoration of articular surgace; and (**c**) fixation of fracture with two 6.5 cannulated screws

**Fig. 2:**
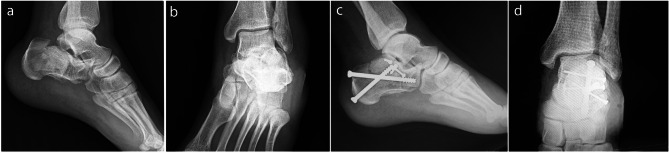
(**a** and **b**) Preoperative anteroposterior and lateral radiographs of the ankle; (**c** and **d**) anteroposterior and lateral radiographs of the ankle immediately after screw fixation

**Fig. 3:**
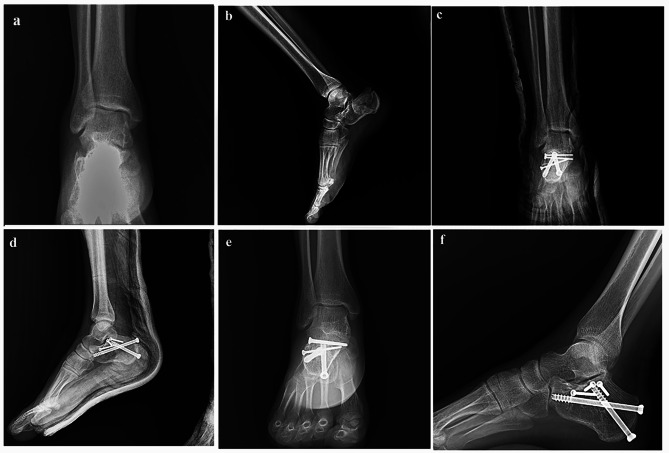
(**a** and **b**) Preoperative anteroposterior and lateral radiographs of the ankle; (**c** and **d**) anteroposterior and lateral radiographs of the ankle immediately after screw fixation; (**e** and **f**) anteroposterior and lateral radiographs of the ankle one year after screw fixation

**Fig. 4:**
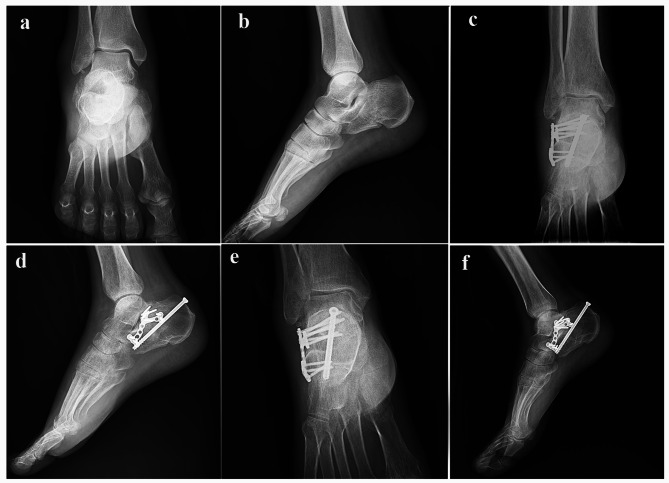
(**a** and **b**) Preoperative anteroposterior and lateral radiographs of the ankle; (**c** and **d**) anteroposterior and lateral radiographs of the ankle immediately after plate fixation; (**e** and **f**) anteroposterior and lateral radiographs of the ankle immediately after plate fixation

Before 2020, the fractures were managed with plate fixation. After 2020, screw fixation was done for all fractures. All the surgeries were done by one senior orthopedic surgeon and in the same center. Patients were placed in the lateral position with the injured side facing up. Under general or spinal anesthesia, the fracture was exposed and managed through an STA, as previously described [13]. After the reduction of the displaced fragments, two or three 2.7 mm screws were inserted below the posterior talocalcaneal joint from lateral to the medial and lagged the posterior facet fragments together. We used three screws for fractures with anterior facet depression or remarkable posterior facet comminution. Then, we made one posterior incision below the Achilles tendon insertion site, and one 6.5 mm cannulated screw was inserted through this incision from the posterior to the anterosuperior under the posterior talocalcaneal joint and one another 6.5 cannulated screw from the posterior to the anterior part of the calcaneus. Appropriate position and length of all implants, heel length, height, width, Bohler’s angle, and Guisan’s angle were confirmed by lateral and axial fluoroscopy (Figs. 1, 2 and 3). The same surgical approach was used in the plate fixation group. We used plates specifically designed for a sinus tarsi approach [14] (Fig. 4). Fluoroscopic examination for appropriate reduction and fixation was done in the same manner as the screw fixation method.

### Postoperative protocol

Immobilization with ankle foot orthosis was done after the operation for one week. Ankle range of motion started one week after the operation. Partial weight bearing was started three weeks after the removal of AFO. Twelve weeks of surgery, fracture healing was assessed radiographically. If fracture healing was observed, full weight-bearing exercises and ambulation were allowed.

### Outcome measures

The outcomes of the surgery were evaluated clinically and radiographically. Clinical evaluation was done in the last follow-up and included the assessment of pain and function. The calcaneal pain was assessed with a Visual Analogue Scale (VAS) rating scale. Accordingly, the patient’s pain was rated on a 0 to 10 scale, indicating no pain and extreme pain, respectively. The calcaneal function was scored using the American Orthopaedic Foot and Ankle Society (AOFAS) Ankle-Hindfoot questionnaire and Foot Function Index (FFI), both ranging from 0 to 100. The minimum score (0) was indicative of no pain or difficulty, while the maximum score stated worst pain and extreme difficulty requiring assistance. Radiographic evaluation of outcomes included the assessment of Bohler’s and Gissane’s angles, and calcaneal length and height on plain lateral radiographs. Radiographic measures were evaluated before the operation, immediately after the operation, and at the time of union. Radiographs were reviewed by two orthopedic residents, and disagreements were resolved by a senior orthopedic surgeon. Postoperative complications were also extracted from the patient’s medical profiles.

### Statistical analysis

SPSS for Windows, version 16 (SPSS Inc., Chicago, Ill., USA) was used for statistical analyses. Descriptive data were demonstrated with mean ± standard deviation (SD) for quantitative variables and numbers with percentages for qualitative variables. The distribution of quantitative variables was checked with a Kolmogorov-Smirnov test, and accordingly, an independent t-test or a Mann–Whitney U test was used to compare quantitative variables between the two groups. Comparison of proportions was made using a chi-squared test and Fisher’s exact test, as appropriate. *P* < 0.05 was considered statistically significant.

## Results

### Baseline characteristics

One hundred and eighty-seven patients with a mean age of 42.6 ± 11.5 years (range 20–79) were included in this study. The study population included 170 (90.9%) males and 17 females (9.1%). The mean follow-up of the patients was 32 ± 9.5 months (range 12–49). No significant difference was observed between the baseline characteristics of the two study groups (Table 1).

**Table 1:** Comparison of baseline characteristics between the two study groups

Variable	Plate fixation group (*n* = 81)	Screw fixation group (*n* = 106)	*P*-value
**Age (year)**	43.4 ± 12	42.1 ± 11.2	0.46
Sex			
• Male • Female	72 (88.9) 9 (11.1)	98 (92.5) 8 (7.5)	0.41
Side			
• Right • Left	37 (45.7) 44 (54.3)	39 (36.8) 67 (63.2)	0.22
Smoking			
• Yes • No	29 (35.8) 52 (64.2)	36 (34) 70 (66)	0.79
Diabetes mellitus			
• Yes • No	3 (3.7%) 78 (94.3)	4 (3.8) 102 (96.2)	0.98
Sanders class			
• II • III	32 (39.5) 49 (60.5)	45 (42.5) 61 (57.5)	0.68
Time interval between injury and surgery (day)	6.6 ± 2.7	6.4 ± 2.5	0.66
Follow-up (month)	42.1 ± 10.5	24.3 ± 8.8	0.001

Date are demonstrated with mean ± SD or number (%). *P* < 0.05 is considered significant

### Radiographic assessment

The radiographic measures of different time points are demonstrated in Table 2. The mean improvement of Bohler’s angle was 13.5 ± 8.1º in the plate fixation group and 16.4 ± 7.9º in the screw fixation group. Accordingly, the mean improvement of Bohler’s angle was significantly greater in the screw fixation group (*P* = 0.014). The mean improvement of the Gissane’s angle was 27.1 ± 13.1º in the plate group and 24 ± 11.7º in the screw fixation group. This difference was not statistically significant (*P* = 0.09). Likewise, improvement in calcaneal length and height were not significantly different between the two study groups (*P* = 0.78 and *P* = 0.22, respectively).

**Table 2:** Change of radiographic measures over time in the two study groups

Variable	Plate fixation group (*n* = 81)	Screw fixation group (*n* = 106)
Bohler’s angle (º)		
• Preoperative • Early postoperative • At the time of union	11.5 ± 6.3 27.3 ± 5.5 25.3 ± 6.1	10.5 ± 6.1 28.6 ± 6 26.7 ± 5.8
Gissane’s angle		
• Preoperative • Early postoperative • At the time of union	157.9 ± 10.7 128.9 ± 7.1 130.1 ± 7.5	154.7 ± 10.5 129.3 ± 7.2 130.4 ± 7.3
Calcaneal length (mm)		
• Preoperative • Early postoperative • At the time of union	90.8 ± 5 85.8 ± 2.4 86.9 ± 4.5	90.2 ± 4.9 86.4 ± 3.8 86.7 ± 4.1
Calcaneal height (mm)		
• Preoperative • Early postoperative • At the time of union	22.2 ± 2.7 32.4 ± 2.9 32.5 ± 3.1	22.2 ± 2.9 32.8 ± 2.1 32.8 ± 2.8

Data are demonstrated with mean ± SD

### Clinical assessments

In the last follow-up, the mean VAS of the patients was 4.2 ± 2.5 in the plate fixation group and 3.2 ± 2.5 in the screw fixation group. This difference was statistically significant (*P* = 0.01). The mean AOFAS score was 78.4 ± 9.5 in the plate fixation group and 80.2 ± 8.7 in the screw fixation group. This difference was not statistically significant (*P* = 0.17). Also, the mean difference in FFI score was not statistically significant between the two study groups (*P* = 0.19). Clinical and radiographic measures are summarized in Table 3.

**Table 3:** Comparison of clinical and radiographic measures between the two study groups

Variable	Plate fixation group (*n* = 81)	Screw fixation group (*n* = 106)	*P*-value
VAS	4.2 ± 2.5	3.2 ± 2.5	0.01
AOFAS Ankle-Hindfoot Score	78.4 ± 9.5	80.2 ± 8.7	0.17
FFI	35.5 ± 21.3	32.1 ± 23.8	0.19
Improvement of Bohler’s angle (º)	13.8 ± 8.3	16.2 ± 8	0.018
Improvement of Gissane’s angle (º)	27.8 ± 13.3	24.3 ± 11.5	0.011
Improvement of Calcaneal length (mm)	3.9 ± 2.9	3.5 ± 3.2	0.53
Improvement of Calcaneal height (mm)	10.3 ± 3.5	10.6 ± 3.7	0.36

VAS: visual analogue scale; AOFAS: American Orthopedic Foot and Ankle Society; FFI: Foot Function Index

Data are demonstrated with mean ± SD or number (%). *P* < 0.05 is considered significant

### Complications

Minor wound complications, including wound dehiscence and superficial infection, occurred in five (6.2%) patients of the plate fixation group and three (2.8%) patients of the screw fixation group. This difference was not statistically significant (*P* = 0.28). Deep infection occurred in three (3.7%) patients of the plate fixation group and no patients of the screw fixation group. This difference was statistically significant (*P* = 0.047). Twelve (14.8%) patients required hardware removal in the plate fixation group versus four (3.8%) patients in the screw fixation group. This difference was also statistically significant (*P* = 0.007).

## Discussion

In this study, we compared the outcomes of a modified screw fixation technique with plate fixation in DIACF managed via an STA. The foot function was not significantly different in the two study groups. However, the pain level was significantly smaller in the screw fixation group. Improvement of Bohler’s angle was also significantly greater in the screw fixation group. However, the improvement of Gissane’s angle, calcaneal length, and height were not significantly different in the two study groups. The patients in the screw fixation group had a significantly lower rate of deep infection and hardware removal.

The outcomes of plate versus screw fixation through an STA for the treatment of DIACF have been evaluated in a small number of earlier studies, and the superiority of either device over the other one is not clear. Guo et al. compared the outcomes of plate versus screw fixation in 165 DIACF patients who were managed through STA. At a mean follow-up of 44.2 months in the plate group and 47.9 months in the screw group, they found no significant difference in the final VAS, AOFAS Ankle-Hindfoot score, and Olerud and Molander ankle score of the two groups [10]. In the study by Cao et al., the same comparison was made by the inclusion of 77 DIACF patients with a mean follow-up of 27 months. They observed no significant difference in the VAS and AOFAS hindfoot scores of the plate and screw fixation [11]. Kir et al. evaluated the outcomes of plate and screw fixation in 60 DIACF patients within one year after the operation. Maryland Foot Score was significantly better in the plate fixation group [15]. In the present study, we did not find any significant difference between the clinical function of the plate and screw fixation. However, the pain level of patients was significantly higher in the plate fixation group at the final visit. This difference could be attributed to the implementation of fewer screws in our modified fixation technique. Moreover, unlike plate fixation, screw fixation does not cause peroneal tendon irritation and subfibular impingement. However, this remains a hypothesis, and due to the retrospective nature of this manuscript, we cannot definitively confirm the exact cause of pain in our patients.

Guo et al. reported no significant difference in Bohler’s angle and Gissane’s angle of patients who were managed with plate fixation compared to those who were managed with screw fixation [10]. The study by Pitts et al. also showed no significant difference between Bohler’s and Gissane’s angle of the plate and screw fixation group [16]. Likewise, Cao et al. found no significant difference between the calcaneal length, height, and Gissane’s and Böhler’s angles of the two fixation groups. However, calcaneal widening was significantly smaller in the plate fixation group [14]. Similar results were reported in the study by Kir et al. [15]. In the present study, the improvement of Böhler’s angles was significantly higher in the screw fixation group. This difference could be attributed to the modification of the screw fixation technique in the present study. We used two 2.7 mm screws to incorporate posterior facet segments, then two 6.5 cannulated screws, one for preventing the collapse of the posterior facet and another one for preventing the lengthening of the calcaneus, maintaining its proper alignment and preventing valgus/varus malalignment in the calcaneus. Furthermore, screws are typically inserted parallel to the bone fragments (posteriorly), whereas in plate fixation, screws are usually inserted perpendicular to the bone fragments. This difference in screw direction could be considered another reason why screws might achieve better reduction in the current study. However, it is important to note that while there was a significant difference in the improvement of the Bohler angle, this did not translate into clinically meaningful differences, as no significant variation was observed in clinical scores.

Various rates of postoperative complications has been reported for plate and screw fixation in earlier studies. Pitts et al. reported only three (4.9%) complications in 61 DIACF patients over a mean follow-up of 46.4 months, including one wound complication in the screw fixation group and two wound complications in the plate fixation group [16]. The total incidence of postoperative complications was 6.7% in the plate fixation group and 6.6% in the screw fixation group of the study by Guo et al. Even so, the rate of implant removal was significantly higher in the plate fixation group (61% vs. 43.4%) [10]. The total incidence of complications was 24.4% in the plate fixation group and 6.3% in the screw fixation group of the study by Cao et al. [11]. In contrast, the rate of postoperative complication and reoperation was significantly higher in the screw fixation group of the study by Kir et al. [15]. In the present study, the rate of postoperative complication, mainly deep infection, was significantly higher in the plate fixation group. This rate can be attributed to the higher irritation of soft tissue for the placement of a plate on the calcaneal wall through a small incision. Also, the rate of implant removal was significantly higher in the plate fixation group (14.8% vs. 3.8%). This higher rate of implant removal is probably the consequence of peroneal tendon irritation and subfibular impingement caused by the plate. Some patients in the screw fixation group also needed device removal. In these patients, screw removal was mainly done because of the pain and irritation of the skin behind the heel when wearing shoes caused by the protrusion of the head of the cannulated screws. Therefore, the need for hardware removal in the screw fixation group could be even more reduced by using headless cannulated screws. It is noteworthy that skin complications, particularly skin necrosis, are among the most concerning complications in calcaneal fractures. In this study, no cases of skin necrosis were observed in any patients in either study group.

In a recent study by Eelsing et al., plate fixation was favored over screw fixation due to the improved immediate Böhler’s angles and lower loss of Böhler’s angles during follow-up. Also, their study showed that a lower rate of implant removal could be obtained if anatomical plate fixation was used via the sinus tarsi approach [17]. In the present study, we used a plate specifically designed for a sinus tarsi approachh. Even so, our results favored the introduced modified screw fixation technique.

While our technique of using screws for the fixation of DAIC through an STA demonstrated promising results, it is beneficial to consider alternative methods, such as the modified percutaneous fixation technique described by Baca and Koluman [18]. Their study presents a percutaneous approach that achieves similar outcomes with low complication rates. This method minimizes soft tissue dissection, which potentially reduces the risk of surgical complications and promotes faster recovery times. Given these findings, it would be advantageous to conduct further comparative studies that evaluate both fixation techniques. Such studies could offer a more comprehensive understanding of the relative benefits and drawbacks of each method, ultimately guiding clinicians in selecting the most effective approach for the fixation of DAICF.

Altogether, the result of the present study, consistent with the results of earlier studies, shows a higher rate of postoperative complications and implant removal in plate fixation of DAICF through an STA. However, it should be noted that the choice of implant is not solely dependent on surgeon preference but also on the specific fracture pattern. In some cases with severe comminution, the fracture pattern may necessitate the use of a plate for fixation purposes.

Also, the present study shows that the outcomes of screw fixation could be optimized with a modified pattern of screw placement. Costs of fixation have also been reported to be smaller when the DIACF is managed with screw fixation [10, 16]. Accordingly, the modified screw fixation technique presented in this study can be suggested as the fixation device of choice for the fixation of DIACF to obtain better clinical and radiographic outcomes, to reduce the rate of postoperative complications, and also to ameliorate financial burden.

The present study was not without limitations. The main limitations of the study were its retrospective design with its potential sources of bias, such as the unequal follow-up for the two study groups. Also, the retrospective design did not allow evaluation of calcaneal widening, which is regarded as a critical factor in the restoration of calcaneal fractures.

## Conclusion

Compared to plate fixation, a modified screw placement pattern using fewer screws could provide lower pain, better radiographic outcomes, and a lower rate of postoperative complications such as deep infection and hardware removal. Therefore, this modified technique can be suggested as the method of choice for DIACF fixation.

## Data Availability

The datasets used and/or analyzed during the current study are available from the corresponding author upon reasonable request.
